# Relation between dry eye and myopia based on tear film breakup time, higher order aberration, choroidal thickness, and axial length

**DOI:** 10.1038/s41598-022-15023-x

**Published:** 2022-06-28

**Authors:** Debabrata Hazra, Erisa Yotsukura, Hidemasa Torii, Kiwako Mori, Tomoki Maruyama, Mamoru Ogawa, Akiko Hanyuda, Kazuo Tsubota, Toshihide Kurihara, Kazuno Negishi

**Affiliations:** 1 Department of Ophthalmology, Keio University School of Medicine, 35 Shinanomachi, Shinjuku-ku, Tokyo, 160-8582 Japan; 2 Laboratory of Photobiology, Keio University School of Medicine, 35 Shinanomachi, Shinjuku-ku, Tokyo, 160-8582 Japan; 3 Tsubota Laboratory, Inc., 34 Shinanomachi, Shinjuku-ku, Tokyo, 160-0016 Japan

**Keywords:** Refractive errors, Corneal diseases

## Abstract

The purpose of this study was to investigate the association between dry eye disease (DED) and myopia by evaluating higher order aberrations (HOAs) and choroidal thickness (CT). We recruited 72 myopic children with DED symptoms (mean age 12.8 years), measured the tear film breakup time (TBUT), corneal/intraocular/total ocular HOAs, CT, and axial length (AL), administered lifestyle questionnaires, and evaluated the relationships among TBUT, HOAs, CT, and AL. The TBUT was correlated significantly with the corneal HOAs and intraocular HOAs but not with the total ocular HOAs. Multiple regression analyses showed that the AL was associated significantly with the TBUT (β = − 0.067, P = 0.004), the intraocular HOAs, and total ocular HOAs but not with the corneal HOAs. The CT was associated significantly with the TBUT and AL (β = 9.15 and − 7.85, respectively; P < 0.001 and = 0.01, respectively). Our data suggested the association between DED and myopia might be independent of the HOAs. We showed that the TBUT was associated with the CT, which is related to the AL. Because the parasympathetic nervous system affects the lacrimal glands and CT, the parasympathetic nervous system might be a common upstream factor in the association between DED and myopia.

## Introduction

Myopia, which increases the risk of vision loss^1^ and deterioration of the mental health of affected individuals^2^, has become a pandemic^3^. It has been estimated that the myopic population will reach about 5 billion in 2050^4^. Identifying factors that affect the occurrence and development of myopia is essential for elucidating the mechanism of myopia and developing reasonable preventative measures or treatments^3^. Previous studies have suggested that myopia is associated with the time spent engaged in outdoor activities^5–9^ and performing near work^10,11^, parental history of myopia^5–7^, and higher order aberrations (HOAs)^5,12–15^.

Regarding the relationships between myopia and HOAs, no consensus has been reached^12^. Some studies have reported lower HOAs in myopic patients^16^, whereas others have reported higher HOAs in myopic patients^17^; the study populations of those studies were around 100 patients^12,16,17^. Recently, multiple regression analysis in our study (n = 1416) showed that the axial length (AL) and refraction were related to the total ocular sum of the 3rd- to 6th-order aberrations (THOA). Briefly, as the THOAs became larger, the ALs and refractions became longer and more myopic, respectively^5^.

Dry eye disease (DED) is a multifactorial disease of the ocular surface^18^ and its prevalence generally increases with age^19^. However, increased use of digital devises such as smartphones especially among adolescents and schoolchildren has increased the risk of DED in younger generations^20,21^. Multiple regression analysis in our previous study showed that the AL and refraction were associated with DED evaluated by a questionnaire^5^. Thus, because we hypothesized that there is a relationship between DED and myopia, we quantified those conditions in the current study. The increased corneal and total ocular HOAs in patients with DED have been reported^22,23^ and the previous studies have described the relationships between HOAs and myopia^5,12,16,17^. We also reported the association between DED and myopia in our previous study^5^. To investigate the relationship between DED and myopia, we must consider whether HOA is a confounder. Therefore, we performed all measurements, i.e., fluorescein tear film breakup time (TBUT), HOAs, and cycloplegic refraction/axial length simultaneously in the current study.

Several treatments are available for myopia progression, one of which is atropine eye drops^24–26^. Although the mechanism of the drop is unknown, the prevention of childhood myopia with 0.01% atropine eye drops preferentially affected subjects with a smaller mesopic pupil diameter, which means a parasympathetic tone-dominant state^26^. Therefore, there seems to be a relationship between the parasympathetic nervous system and myopia. The choroid, which is involved in the modulation of ocular elongation and control of the refractive error^27^, and the lacrimal glands^28^ are both innervated by the parasympathetic nervous system; the choroidal thickness (CT) is correlated negatively with the AL^29–31^. In the current study, we measured the CT, AL, and TBUT, because we hypothesized that the parasympathetic nervous system may be a common upstream factor in both the TBUT and CT, so dry eye and myopia may be downstream results. In the current study, we report the relationships among the TBUT, HOAs, CT, and AL.

## Results

### Patient demographics

The mean age of the 72 patients (boys 61.1%; girls 38.9%) was 12.8 ± 2.7 (standard deviation [SD]) years (range 4–16 years). The mean TBUT, CT, AL, and SE of the participants were, respectively, 5.7 ± 3.1 s (1–10 s), 285.3 ± 38.4 μm (205–375 μm), 25.52 ± 1.14 mm (21.36–27.82 mm), and − 4.61 ± 2.40 D (− 10.33 to − 0.59 diopter) (Table 1). Our results showed that 94.9% of the myopic children had some symptoms of DED, and 73.6% and 54.7% of participants had TBUTs of < 10 and ≤ 5 s, respectively. Participants who had some DED symptoms and TBUT values of ≤ 5 s and those who had either severe DED symptoms or a history of clinically diagnosed DED evaluated by questionnaire^32–34^ accounted for 51.9% and 67.9%, respectively.

**Table 1:** Subject demographic data.

Parameter	Number	Mean ± SD (range)
Age (years)	72	12.8 ± 2.7 (4 to 16)
Gender	72	Boy N = 44 (61.1%), Girl N = 28 (38.9%)
Fluorescein TBUT (seconds)	53	5.7 ± 3.1 (1 to 10)
Choroidal thickness (μm)	70	285.3 ± 38.4 (205 to 375)
Axial length (mm)	72	25.52 ± 1.14 (21.36 to 27.82)
Cycloplegic objective refraction (diopters)	37	− 4.61 ± 2.40 (− 10.33 to − 0.59)
Non-cycloplegic objective refraction (diopters)	72	− 4.57 ± 2.23 (− 10.35 to − 0.65)
UCVA (logMAR)	72	0.94 ± 0.35 (− 0.08 to 1.52)
BCVA (logMAR)	72	− 0.08 ± 0.00 (− 0.08 to − 0.08)

Data are expressed as the mean ± standard deviation. *SD* standard deviation, *TBUT* tear film breakup time, *UCVA* uncorrected visual acuity, *BCVA* best-corrected visual acuity, *logMAR* logarithm of the minimum angle of resolution. The respective number of cases are shown.

### Corneal HOAs, intraocular HOAs, and total ocular HOAs

Supplementary Table 1 shows the corneal/intraocular/total ocular SA, S3, S4, and THOA values.

### Associations between myopia and lifestyle factors

Table 2 shows the results of multiple regression analyses to identify the relationships between AL, cycloplegic objective refraction, and lifestyle factors including the TBUT using the questionnaires. Significant correlations were found between the AL and TBUT (β = − 0.067, *P* = 0.004), age (β = 0.065, *P* = 0.02), parental history of myopia (β = 0.497, *P* = 0.04), and time spent outdoors (β = − 0.027, *P* < 0.001). However, no significant correlations were found between cycloplegic objective refraction and any lifestyle factors including the TBUT (Table 2).

**Table 2:** Results of multiple regression analyses to estimate the association between myopia and environmental factors.

	Axial length, mm (N = 53)			Cycloplegic objective refraction, diopter (N = 24)		
	β	95% CI	*P*	β	95% CI	*P*
**Overall**						
Fluorescein TBUT (seconds)	− 0.067	− 0.110 to − 0.023	**0.004**	0.149	− 0.195 to 0.492	0.41
Age (years)	0.065	0.012 to 0.118	**0.02**	0.227	− 0.308 to 0.762	0.42
Gender	− 0.102	− 0.381 to 0.178	0.48	0.658	− 1.75 to 3.07	0.60
**Number of myopic parents**						
None	NA	NA	NA	NA	NA	NA
One	0.242	− 0.238 to 0.723	0.33	− 1.14	− 3.81 to 1.53	0.43
Both	0.497	0.033 to 0.960	**0.04**	− 4.10	− 8.590 to 0.385	0.10
Outdoor activity time (min/day)	− 0.027	− 0.034 to − 0.021	** < 0.001**	0.012	− 0.047 to 0.072	0.69
Sleeping time (min/day)	0.002	− 0.0002 to 0.004	0.08	0.010	− 0.007 to 0.028	0.26
Near work time (min/day)	0.001	− 0.001 to 0.003	0.33	− 0.009	− 0.021 to 0.003	0.15
Reading distance (cm)	− 0.011	− 0.033 to 0.011	0.36	− 0.069	− 0.222 to 0.085	0.40
R^2^		0.831			0.508	

Two multiple regression models were used. Axial length or cycloplegic objective refraction was used as the outcome variable. *P* values less than statistically significant level (< 0.05) are marked in bold. For gender, boys were numbered 1 and girls were numbered 0. *β* coefficient, *95% CI* 95% confidence interval, *TBUT* tear film breakup time, *NA* not applicable.

### Correlations between TBUT and HOAs

Supplementary Figs. 1, 2, and 3 show the correlations and the Spearman's rank correlation coefficients (r) of the TBUT with the corneal, intraocular, and total ocular HOAs. Significant correlations were found between the TBUT and corneal HOAs (TBUT vs. corneal HOAs: SA, r = − 0.323, *P* = 0.02; S4, r = − 0.497, *P* < 0.001; THOA, r = − 0.362, *P* = 0.009) (Supplementary Fig. 1) and the intraocular HOAs (TBUT vs. intraocular HOAs: S3, r = − 0.299, *P* = 0.04; S4, r = − 0.369, *P* = 0.008; THOA, r = − 0.368, *P* = 0.009) (Supplementary Fig. 2). However, no significant correlations were found between the TBUT and total ocular HOAs (Supplementary Fig. 3).

### Associations between myopia and HOAs

Table 3 shows that multiple regression analyses did not find significant correlations between the AL, refraction, and corneal HOAs.

**Table 3:** Results of multiple regression analyses to estimate the association between myopia and corneal higher order aberrations (evaluated with natural pupillary diameters, average value φ = 6.1 mm).

	Axial length, mm (N = 59)			Cycloplegic objective refraction, D (N = 25)		
	β	95% CI	*P*	β	95% CI	*P*
**Overall**						
Age (years)	0.176	0.073 to 0.279	**0.001**	0.142	− 0.323 to 0.606	0.56
Gender	0.258	− 0.320 to 0.836	0.39	− 0.795	− 2.69 to 1.10	0.42
SA (μm)	− 0.633	− 3.26 to 1.99	0.64	10.2	− 27.3 to 47.8	0.60
S3 (μm)	− 11.2	− 32.8 to 10.4	0.32	11.3	− 71.3 to 93.9	0.79
S4 (μm)	− 3.63	− 29.9 to 22.7	0.79	− 36.9	− 202 to 128	0.67
THOA (μm)	9.75	− 19.0 to 38.5	0.51	5.98	− 109 to 121	0.92
R^2^		0.294			0.421	

*D* diopters, *SA* spherical aberration, *S3* 3rd-order aberrations, *S4* 4th-order aberrations, *THOA* sum of the 3rd- to 6th-order aberrations, *95% CI* 95% confidence interval, *β* coefficient.

Two multiple regression models were used. Axial length or cycloplegic objective refraction was used as the outcome variable. *P* < 0.05 are highlighted. For gender, boys were numbered 1 and girls were numbered 0.

Table 4 shows that significant correlations were found between the AL and the intraocular S3 (β = − 21.8, *P* = 0.02) and the intraocular THOA (β = 21.2, *P* = 0.048) and between the cycloplegic refraction and the intraocular S3 (β = 96.9, *P* = 0.04) and intraocular THOA (β = − 112, *P* = 0.049).

**Table 4:** Results of multiple regression analyses to estimate the association between myopia and intraocular higher order aberrations (evaluated with natural pupillary diameters, average value φ = 6.1 mm).

	Axial length, mm (N = 59)			Cycloplegic objective refraction, D (N = 25)		
	β	95% CI	*P*	β	95% CI	*P*
**Overall**						
Age (years)	0.183	0.086 to 0.280	** < 0.001**	0.314	− 0.213 to 0.842	0.26
Gender	0.178	− 0.375 to 0.731	0.53	− 0.910	− 3.03 to 1.21	0.41
SA (μm)	1.53	− 0.473 to 3.52	0.14	− 5.06	− 29.2 to 19.1	0.69
S3 (μm)	− 21.8	− 40.0 to − 3.55	**0.02**	96.9	11.6 to 182	**0.04**
S4 (μm)	− 9.08	− 24.2 to 6.04	0.24	58.3	− 50.7 to 167	0.31
THOA (μm)	21.2	0.634 to 41.8	**0.048**	− 112	− 221 to − 2.65	**0.049**
R^2^		0.365			0.285	

*D* diopters, *SA* spherical aberration, *S3* 3rd-order aberrations, *S4* 4th-order aberrations, *THOA* sum of the 3rd- to 6th-order aberrations, *95%* *CI* 95% confidence interval, *β* coefficient Two multiple regression models were used. Axial length or cycloplegic objective refraction was used as the outcome variable. *P* < 0.05 are highlighted. For gender, boys were numbered 1 and girls were numbered 0.

Table 5 shows the significant correlations between the AL and the total ocular S3 (β = − 41.7, *P* = 0.045) and the total ocular THOA (β = 43.6, *P* = 0.049). No significant correlations were found between the refraction and total ocular HOAs.

**Table 5:** Results of multiple regression analyses to estimate the association between myopia and total ocular higher order aberrations (evaluated with natural pupillary diameters, average value φ = 6.1 mm).

	Axial length, mm (N = 59)			Cycloplegic objective refraction, D (N = 25)		
	β	95% CI	*P*	β	95% CI	*P*
**Overall**						
Age (years)	0.171	0.070 to 0.273	**0.002**	0.126	− 0.413 to 0.665	0.65
Gender	0.244	− 0.350 to 0.839	0.42	− 0.875	− 3.15 to 1.40	0.46
SA (μm)	− 0.123	− 3.16 to 2.91	0.94	3.03	− 6.20 to 12.3	0.53
S3 (μm)	− 41.7	− 81.7 to − 1.79	**0.045**	106	− 89.4 to 302	0.30
S4 (μm)	− 23.8	− 50.2 to 2.60	0.08	61.5	− 78.2 to 201	0.40
THOA	43.6	0.954 to 86.3	**0.049**	− 119	− 332 to 94.3	0.29
R^2^		0.278			0.155	

*D* diopters, *SA* spherical aberration, *S3* 3rd-order aberrations, *S4* 4th-order aberrations, *THOA* sum of the 3rd- to 6th-order aberrations, *95% CI* 95% confidence interval, *β* coefficient. Two multiple regression models were used. Axial length or cycloplegic objective refraction was used as the outcome variable. P < 0.05 are highlighted. For gender, boys were numbered 1 and girls were numbered 0.

### Associations between the CT and TBUT, AL

**Figure 1:**
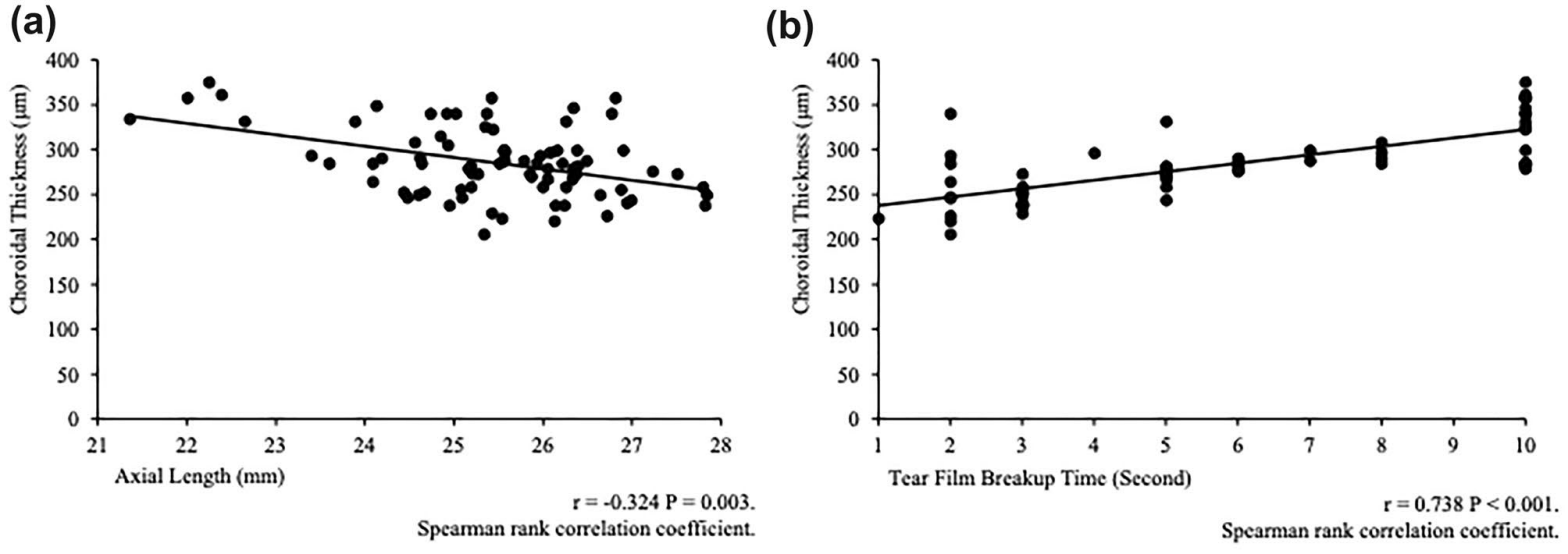
The correlations between the choroidal thickness (CT) and the tear film breakup time (TBUT) and axial length (AL). (**a**) The correlations between the CT and the TBUT; and (**b**) the CT and the AL. The Spearman rank correlation coefficients and *P* values are shown between the CT and the TBUT and between the CT and the AL. Significant correlations are seen between the CT and the TBUT and AL.

Figure 1 shows the significant correlations between the CT and TBUT (r = 0.738 *P* < 0.001) (Fig. 1a) and between the CT and AL (r = − 0.324 *P* = 0.003) (Fig. 1b).

Table 6 shows the significant correlations between the CT and gender (β = 18.1, *P* = 0.01), TBUT (β = 9.15, *P* < 0.001), and AL (β = − 7.85, *P* = 0.01).

**Table 6:** Results of multiple regression analyses to estimate the association between choroidal thickness and dry eye disease.

	Choroidal thickness, μm (N = 52)		
	β	95% CI	*P*
**Overall**			
Age (years)	− 2.13	− 4.67 to 0.405	0.11
Gender	18.1	4.29 to 31.9	**0.01**
Fluorescein TBUT (seconds)	9.15	7.09 to 11.2	** < 0.001**
Axial length (mm)	− 7.85	− 13.9 to − 1.83	**0.01**
R^2^		0.662	

A multiple regression model was used. Choroidal thickness was used as the outcome variable. *P* < 0.05 are highlighted. For gender, boys were numbered 1 and girls were numbered 0. *β* coefficient, *95% CI* 95% confidence interval, *TBUT* tear film breakup time.

## Discussion

**Figure 2:**
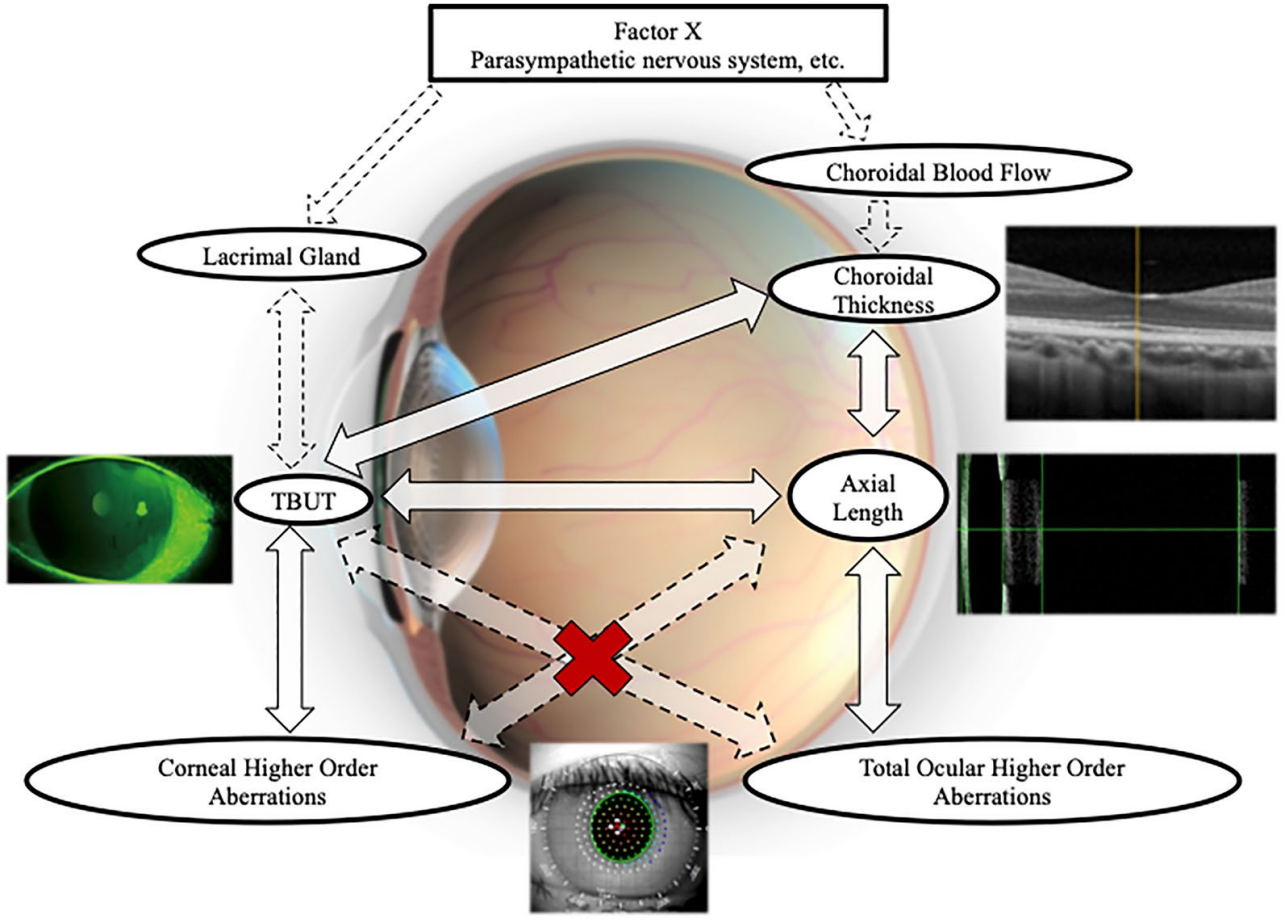
The schema of the relationships among the tear film breakup time (TBUT), higher order aberrations (HOAs), choroidal thickness (CT), and axial length (AL) from the results of the current study. The TBUT is associated significantly with the CT that is related to the AL. The TBUT is correlated with the corneal HOAs but not with the total ocular HOAs, whereas the AL is related to the total ocular HOAs but not to the corneal HOAs. Because the parasympathetic nervous system affects both the lacrimal glands and CT, the parasympathetic nervous system might be a common factor upstream in the association between the TBUT and the CT.

The current study investigated the relationships among the TBUT, HOAs, CT, and AL. We confirmed a quantitative relationship between myopia and DED by evaluating the TBUT; the AL increased as the TBUT became shorter. The results also showed that the TBUT was correlated with the corneal HOAs and CT but not with the total ocular HOAs, and the AL was related to the total ocular HOAs and CT but not to the corneal HOAs. The relationships among the TBUT, HOAs, CT, and AL in the current study are summarized in Fig. 2. Our results suggested that the TBUT is related to the CT and that the association between the TBUT and AL might be independent of the HOAs.

Multiple studies have investigated the relationships between myopia and HOAs^5,12–15^. Philip et al.^13^ indicated that the risk of becoming myopic increased as the total ocular SA decreased. Lau et al.^14^ also reported that the amount of axial elongation decreased as the total ocular SA and total ocular THOA increased after adjusting for age, sex, and refractive error. However, Zhang et al.^15^ reported that total ocular coma aberration, total ocular S3, and total ocular THOA were higher in children in whom myopia progressed rapidly (change of SE, > 0.5 D/year).

Although all of the previously mentioned studies have considered only total ocular HOAs^5,12–15^, a recent study of 64 myopic Japanese children (mean age 9.2 ± 1.6 year, under cycloplegia) in whom the corneal HOAs and total ocular HOAs were measured showed that the corneal THOA was the variable that was most relevant to myopia progression and axial elongation among schoolchildren^35^. The current study found that the AL was related to intraocular HOAs and total ocular HOAs. Contradictions between the current and previous study^35^ in which DED was not evaluated might be attributed to the consideration of DED. The current study found significant correlations between the TBUT and corneal/intraocular HOAs. Thus, we did not include the TBUT as an independent variable in multiple regression analyses that examined the associations between myopia and HOAs because of the multicollinearity and found that the AL and refraction were associated with intraocular HOAs but not with corneal HOAs. Together with the previous study^35^, our data suggested that DED was a confounder in the associations between myopia and HOAs.

To date, few studies have evaluated the relationship between DED and myopia^5,36^. Ilhan et al.^36^ reported that adult patients with high myopia (SE < − 6.0 D) had a higher incidence of DED. Our previous pediatric study also showed that DED assessed by questionnaires was correlated with the AL and non-cycloplegic refraction^5^. In the current study, we quantified the DED severity by measuring the TBUT and examined its relationships with AL and cycloplegic refraction. The prevalence of DED in this study (51.9%) was higher than that of childhood DED (mean age 15.2 ± 5.6 years) reported previously (0.4%)^37^. Our results indicated a quantitative relationship between the TBUT and AL, suggesting myopia may be related to at least one measure of DED.

The corneal HOAs and total ocular HOAs were elevated in DED^22,23,38,39^, especially in DED with a short TBUT^40^, due to irregular astigmatism in the tear film^41^. However, all previous studies included adults^22,23,38,39^, and the associations between HOAs and DED among children have yet to be investigated. In the current study, we showed that corneal HOAs but not total ocular HOAs increased as the TBUT shortened in children. Surprisingly, the intraocular HOAs also became elevated as the TBUT shortened in the current study. Because we evaluated the HOAs with the RMS value, we could not judge the positivity or negativity. The intraocular SA, which was not evaluated with the RMS value, had the opposite correlation tendency toward the TBUT compared to the corneal SA. This indicated that the signs of the corneal HOAs and intraocular HOAs might be opposite, and the corneal HOAs related to DED might be compensated for by the intraocular HOAs.

To the best of our knowledge, this is the first study in which the CT was associated significantly with the TBUT. Previous studies have reported the crucial role of the choroid in the modulation of ocular elongation and homeostatic control of the refractive error^27^. Parapapillary diffuse choroidal atrophy, which involves extreme thinning of parapapillary choroid^42^, is a potential precursor of pathologic myopia^43^. The CT is correlated negatively with the AL in patients with high myopia^29,30^ and healthy subjects^31^ Our results also showed that the CT is correlated negatively with the AL. We can suggest two plausible explanations for the association between the TBUT and CT. First, since increased parasympathetic tone enhances both the CT^27^ and the amount of tear secretion from the lacrimal glands^28^, our results suggested that a “factor X”, including the parasympathetic nervous system, might be a common factor upstream in the association between the TBUT (DED) and CT (myopia) (Fig. 2). Another explanation might be simpler, i.e., those who perform near work have a greater increased risk of both evaporative DED, which directly affects the TBUT, and myopia, which affects the CT.

The current study had limitations. First, because it was not a longitudinal study, it could not establish causality. Second, the sample size was small. Third, selection bias was possible because the study included only those who visited our clinic. Fourth, we cannot directly evaluate the lacrimal gland function based on the TBUT and should perform other tests such as the Schirmer’s test or the phenol red test to directly evaluate secretion from the lacrimal glands in a future study.

## Materials and methods

### Study design and study populations

This cross-sectional study adhered to the tenets of the Declaration of Helsinki and was approved by the Keio University School of Medicine Ethics Committee (approval number: 20180189). Since ethical guidelines for clinical studies by Japanese Ministry of Health, Labor and Welfare indicate that researchers do not need to obtain written informed consent from each patient for studies not involving biologic tissue but only to review the medical records retrospectively, we displayed our ethical statement and written guidelines for the current study on our website in Japanese^44^. The requirement for obtaining written informed consent was waived by the Keio University School of Medicine Ethics Committee.

The inclusion criterion was presentation to the Keio University Hospital Myopia Clinic from July 2017 to December 2019. A total of 84 consecutive patients participated during that period and none had been treated with atropine or underwent any ocular surgery. The exclusion criteria included either hyperopia or emmetropia (n = 6), best-corrected visual acuity of < 20/20 (n = 2, amblyopia), and the absence of symptoms of either foreign-body sensation or ocular dryness (n = 4). Ultimately, 72 (85.7%) had data available for our study.

### Measurements

We performed slit-lamp examinations to evaluate the fluorescein TBUT of the patients. Patients underwent eye examinations that included measurement of cycloplegic or non-cycloplegic refraction and ocular biometric parameters such as anterior chamber depth, lens thickness, AL, CT, and HOAs. Trained orthoptists performed all examinations. For cycloplegia, 1 drop of 0.4% oxybuprocaine hydrochloride (benoxinate hydrochloride 0.4%, Santen Pharmaceutical, Osaka, Japan) was followed by 1 drop of 0.5% tropicamide and 0.5% phenylephrine hydrochloride (Mydrin-P, Santen Pharmaceutical) and 1% cyclopentolate hydrochloride (Cyplegin 1% ophthalmic solution, Santen Pharmaceutical) administered 5 min apart. The measurements were obtained 60 min after the initial drop instillation. Refractions were measured by autorefractometry (TONOREF III, Nidek, Gamagori, Japan). Ocular biometric parameters were measured by noncontact optical biometry using swept-source optical coherence tomography (OCT) biometry (IOLMaster 700, Carl Zeiss Meditec AG, Jena, Germany), which has a measurement accuracy of ± 5 um. We recorded the AL 10 times and averaged the data. High-resolution OCT (RS-3000 Advance, Nidek) was used to measure the subfoveal CT. We defined CT as mentioned previously^45^ and one clinician manually performed all the measurements using ImageJ software (Rasband WS, ImageJ, National Institutes of Health, Bethesda, MD). All of the CT measurements were performed between 2:00 and 4:00 pm to consider the diurnal variations of the CT. The non-cycloplegic corneal and total ocular HOAs were measured using the HOYA iTrace Surgical Workstation (Tracey Technologies, Houston, TX). The HOAs (μm) were evaluated with a natural pupillary diameter (average value φ = 6.1 mm), and the root mean square (RMS) values from the 3rd- to 6th-order Zernike coefficients were calculated. From these Zernike coefficients, the spherical aberration (SA), 3rd-order aberrations (S3), 4th-order aberrations (S4), and THOA were calculated. Each intraocular HOA was calculated by subtracting the respective corneal HOA from the total ocular HOA. Some patients wore contact lenses for vision correction (n = 13), and we did not measure their TBUT and HOAs and measured the AL, cycloplegic refraction, non-cycloplegic refraction, CT, UCVA, and BCVA when the contact lens were removed.

The patients completed a lifestyle questionnaire that included factors such as time spent outdoors, doing near work, and sleeping; symptoms of DED; and parental history of myopia. We defined time spent outdoors as the average number of hours spent outdoors daily that was calculated using the following formula: [(hours spent on a weekday) × 5 + (hours spent on a weekend day) × 2]/7^46^. Near work included studying; reading books; using a computer, tablet. or smartphone; watching television, and playing video or portable games. The DED symptoms recorded using the Women’s Health Study questionnaire^32^ in Japanese^33,34^.

We defined myopia as a spherical equivalent (SE) of ≤ − 0.5 diopter (D) in the current study; only the results from the left eye are presented.

### Sample size calculation

The sample size calculation indicated that a sample size of at least 48 cases would be required (the minimally acceptable correlation coefficient between CT and BUT was 0.45 [r = 0.45], so 48 cases were needed to have a 5% alpha level and 90% power).

### Statistical analysis

We analyzed the correlations among the TBUT and HOAs, TBUT and CT, and CT and AL by calculating Spearman's rank correlation coefficient. Using multiple regression analysis, we examined the associations between the outcomes (AL and cycloplegic objective refraction) and other factors including age, gender, parental history of myopia, time spent outdoors, time spent on near work, reading distance, sleeping time, and TBUT. We also investigated the associations between outcomes (AL and cycloplegic objective refraction) and other factors including age, gender and HOAs (SA, S3, S4, and THOA) of either the corneal, intraocular or total ocular HOAs by multiple regression analysis. Finally, to evaluate the associations between CT and other factors including age, gender, TBUT, and AL, we performed multiple regression analyses. In all multiple regression analyses, we evaluated the age and gender by forced entry and the other factors by stepwise analysis because age and gender have already been reported as factors associated with myopia^47^.

All statistical analyses were performed using a statistical analysis software (R version 4.0.0, R Foundation for Statistical Computing, Vienna, Austria). All *P* values were considered significant if < 0.05.

## Conclusions

We showed that the TBUT is associated significantly with the CT, which is correlated with the AL. Because the parasympathetic nervous system affects the lacrimal glands and CT, we suggest that the parasympathetic nervous system might be a common factor upstream in the association between DED and myopia. Further study is needed to confirm this hypothesis.

## Supplementary Information

Supplementary Information.

## Data Availability

Debabrata Hazra and Erisa Yotsukura had full access to all the study data and take responsibility for the integrity of the data and the accuracy of the data analysis. The data that support the findings of this study are available from Debabrata Hazra and Erisa Yotsukura but restrictions apply to the availability of these data, which were used under license for the current study, and so are not publicly available. Data are however available from the authors upon reasonable request and with permission of Debabrata Hazra and Erisa Yotsukura.
