# Supplementary material for: Relation between dry eye and myopia based on tear film breakup time, higher order aberration, choroidal thickness, and axial length
Source: Sci Rep. 2022 Jun 28;12:10891. doi: 10.1038/s41598-022-15023-x (PMC9240066; doi:10.1038/s41598-022-15023-x)
Supplement: Supplementary file 1 — Supplementary Information. [file 41598_2022_15023_MOESM1_ESM.pdf]

Relation between Dry Eye and Myopia Based on Tear Film Breakup Time, Higher Order Aberration, Choroidal Thickness, and Axial Length

Debabrata Hazra, MD;[*] Erisa Yotsukura, MD, PhD;[*] Hidemasa Torii, MD, PhD;[*] Kiwako Mori, MD, PhD; Tomoki Maruyama, MD; Mamoru Ogawa, MD, PhD; Akiko Hanyuda, MD, PhD; Kazuo Tsubota, MD, PhD; Toshihide Kurihara, MD, PhD, Kazuno Negishi, MD, PhD

Author Affiliations: Department of Ophthalmology, Keio University School of Medicine, 35 Shinanomachi, Shinjuku-ku, Tokyo 160-8582, Japan (Hazra, Yotsukura, Torii, Mori, Maruyama, Ogawa, Hanyuda, Tsubota, Kurihara, Negishi); Laboratory of Photobiology, Keio University School of Medicine, 35 Shinanomachi, Shinjuku-ku, Tokyo 160-8582, Japan (Hazra, Yotsukura, Torii, Mori, Ogawa, Hanyuda, Kurihara); Tsubota Laboratory, Inc., 34 Shinanomachi, Shinjuku-ku, Tokyo 160-0016, Japan (Tsubota)

Supplementary Figure 1

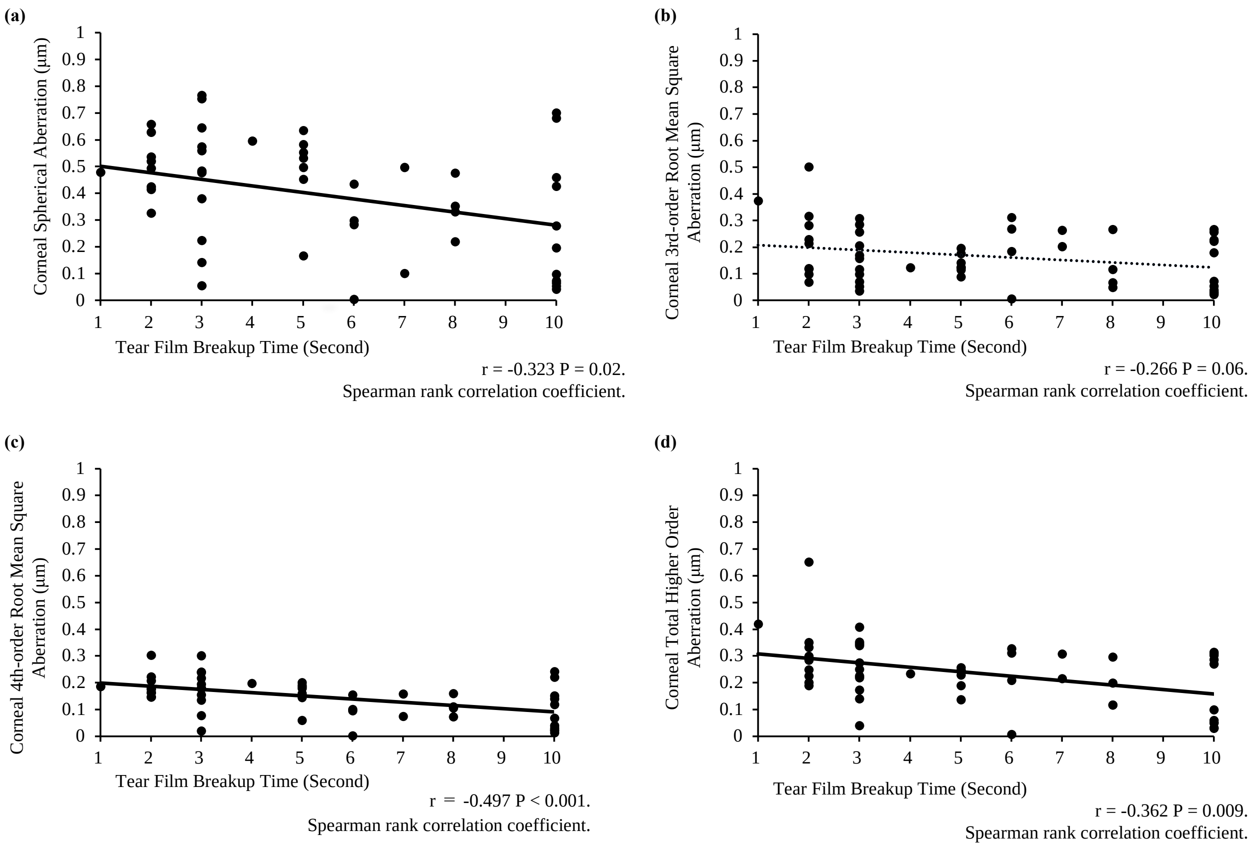

Supplementary Figure 1. Correlations between the tear film breakup time (BUT) and the corneal higher order aberrations (HOAs). (a) The correlations between the BUT and the corneal spherical aberration; **(b)** the BUT and the corneal coma-like aberration; **(c)** the BUT and the corneal spherical-like aberration; **(d)** and the BUT and the corneal total higher order aberration. The Spearman rank correlation coefficients and *P* values are shown between the BUT and each corneal HOA in the eyes of myopic children. Significant correlations are seen between the BUT and the corneal spherical aberration, the corneal spherical-like aberration, and the corneal total higher order aberration.

Supplementary Figure 2

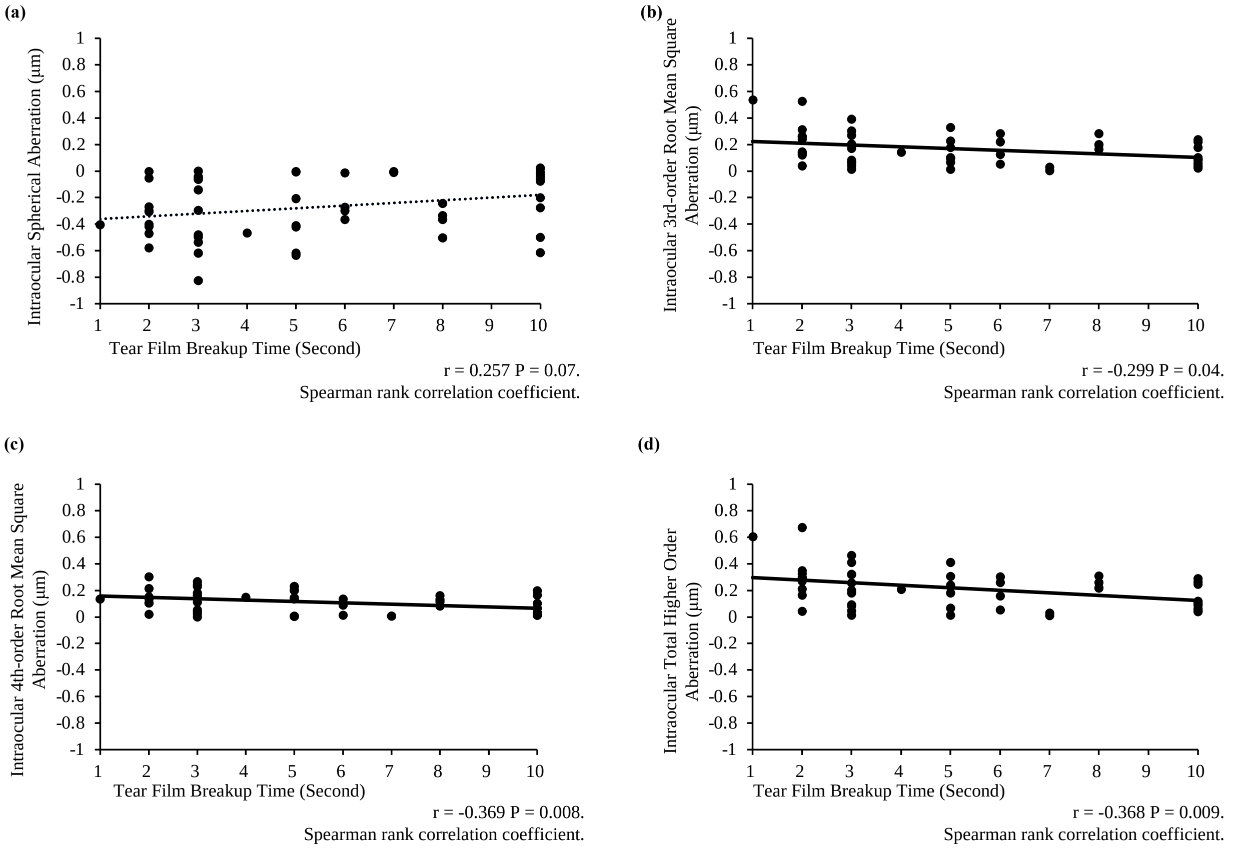

Supplementary Figure 2. The correlations between the tear film breakup time (BUT) and the intraocular higher order aberrations (HOAs). (a) The correlations between the BUT and the intraocular spherical aberration; **(b)** the BUT and the intraocular coma-like aberration; **(c)** the BUT and the intraocular spherical-like aberration; and **(d)** the BUT and the intraocular total higher order aberration. The Spearman rank correlation coefficients and *P* values are shown between the BUT and each intraocular HOA in the eyes of myopic children. Significant correlations are seen between the BUT and the intraocular coma-like aberration, intraocular the spherical-like aberration, and the intraocular total higher order aberration.

Supplementary Figure 3

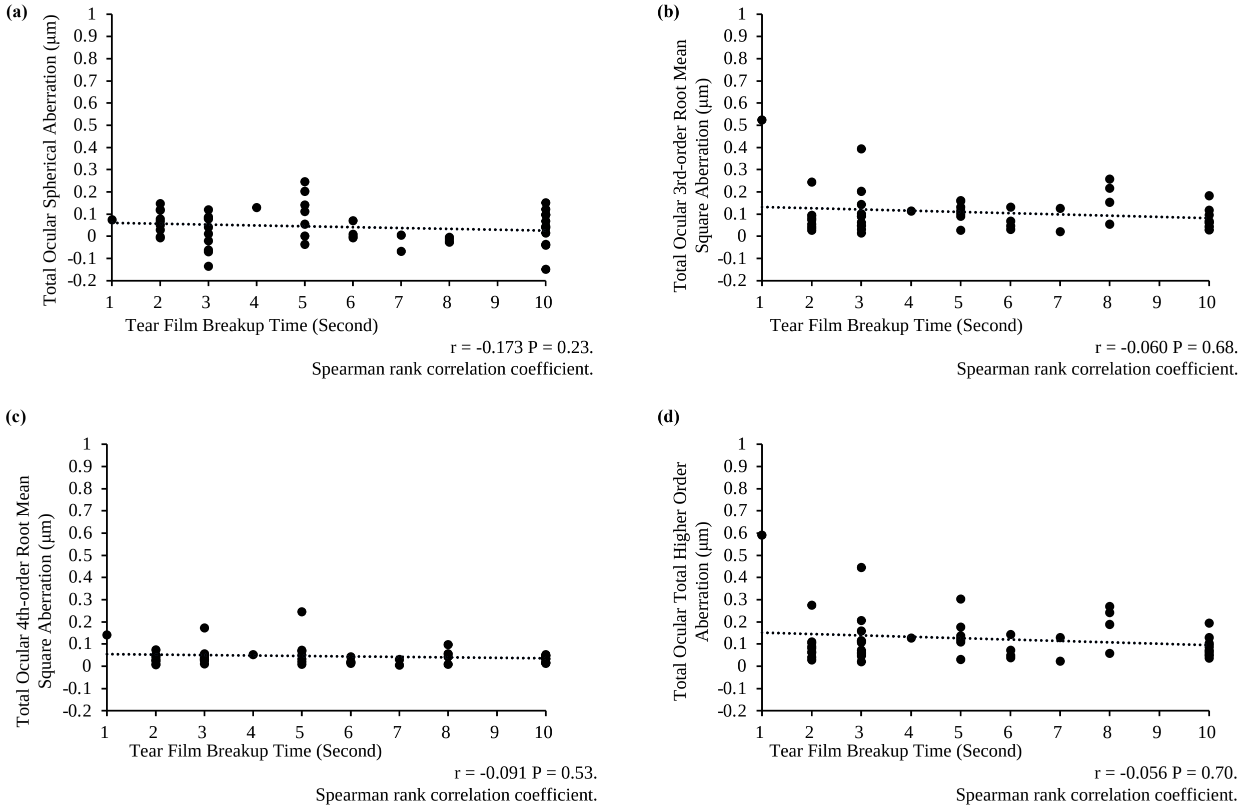

Supplementary Figure 3. The correlations between the tear film breakup time (BUT) and the total ocular higher order aberrations (HOAs). (a) The correlations between the BUT and the total ocular spherical aberration; **(b)** the BUT and the total ocular coma-like aberration; **(c)** the BUT and the total ocular spherical-like aberration; and **(d)** the BUT and the total ocular total higher order aberration. The Spearman rank correlation coefficients and *P* values are shown between the BUT and each total ocular HOA in the eyes of myopic children. No significant correlations are seen between the BUT and any total ocular HOAs.

Supplementary Table 1. Higher-Order Aberrations of Subjects (Evaluated with Natural Pupillary Diameters, Average Value φ = 6.1 mm)

	**Parameter**	**Mean ± SD (Range)**
Corneal	SA, µm	0.3687 ± 0.2178 (-0.0038 ~ 0.7674)
	S3, µm	0.1560 ± 0.1055 (0.0068 ~ 0.5010)
	S4, µm	0.1364 ± 0.0748 (0.0028 ~ 0.3040)
	THOA, µm	0.2188 ± 0.1228 (0.0073 ~ 0.6520)
Intraocular	SA, µm	-0.2599 ± 0.2198 (-0.8220 ~ 0.0415)
	S3, µm	0.1574 ± 0.1190 (0.0063 ~ 0.5383)
	S4, µm	0.1062 ± 0.0778 (0.0017 ~ 0.3053)
	THOA, µm	0.2028 ± 0.1430 (0.0126 ~ 0.6765)
Total ocular	SA, µm	0.0370 ± 0.0906 (-0.3100 ~ 0.2469)
	S3, µm	0.1055 ± 0.0886 (0.0168 ~ 0.5247)
	S4, µm	0.0451 ± 0.0410 (0.0057 ~ 0.2464)
	THOA, µm	0.1226 ± 0.1003 (0.0228 ~ 0.5913)

Data are expressed as the mean ± SD. Number of cases was 59.

SD = standard deviation; SA = spherical aberration; S3 = 3rd-order aberrations’ S4 = means 4th-order aberrations; THOA = sum of the 3rd- to 6th-order aberrations.
